# The Role of Family Influences in Development and Risk

**Published:** 1997

**Authors:** Deborah A. Ellis, Robert A. Zucker, Hiram E. Fitzgerald

**Affiliations:** Deborah A. Ellis, Ph.D., is an assistant professor in the Department of Psychiatry and Behavioral Neurosciences, Wayne State University, Detroit, Michigan. Robert A. Zucker, Ph.D., is professor of psychology in the Departments of Psychiatry and Psychology, University of Michigan, Ann Arbor, Michigan. Hiram E. Fitzgerald, Ph.D., is professor and associate chairperson in the Department of Psychology, Michigan State University, East Lansing, Michigan

**Keywords:** children of alcoholics, family environment, family as an AODC (causes of AOD use, abuse, and dependence), behavioral and mental disorder, AOD use susceptibility, parent, AOD use behavior, ethnic group, expectancy, socioeconomic status, psychological history, dual diagnosis, hereditary factors, family relations, interpersonal interaction, risk factors, cognition, literature review

## Abstract

Various influences in the family environment contribute to children of alcoholics’ (COAs’) risk of developing alcoholism and other mental health problems. These risk factors include alcohol-specific influences, which selectively predict alcohol problems, and alcohol-nonspecific influences, which predict a variety of mental health problems. Alcohol-specific family influences include modeling of parental drinking behavior, development of alcohol expectancies, and the family’s ethnic background. Parental psychopathology, the family’s socioeconomic status, and general family psychopathology are examples of alcohol-nonspecific risk factors, which increase the COA’s risk of behavior disorders as well as of alcoholism. The families of COA’s who are at highest risk for alcoholism and other mental health problems are characterized by the aggregation of numerous alcohol-specific and alcohol-nonspecific risk factors.

Researchers and clinicians have known for some time that children of alcoholics (COA’s) exhibit elevated rates of psychopathology. For example, COA’s are approximately four to six times as likely as the general population to develop alcohol problems^1^ (Russell 1990). Furthermore, anxiety, depression, and externalizing behavior disorders (e.g., conduct disorder) are more common among COA’s than among children of nonalcoholics (non-COA’s) (West and Prinz 1987; Seilhamer and Jacob 1990). Nevertheless, the majority of COA’s show no evidence of significant mental health problems (including alcohol and other drug [AOD] abuse) during adulthood. COA’s therefore can be considered an at-risk population in which the occurrence of significant psychopathology in a given individual results from the convergence of various risk factors at a particular time during development.

One of the most compelling explanations for the wide range in mental health outcomes among COA’s is the significant variability (i.e., heterogeneity) that exists among alcoholic families (i.e., families with at least one alcohol-abusing parent) (Seilhamer and Jacob 1990; Chassin et al. 1991; Zucker et al. 1996*b*). Several studies have found that certain familial characteristics (e.g., comorbid psychiatric disorders in the parents) substantially affect the likelihood that COA’s will suffer maladaptive outcomes (Sher et al. 1991; Finn et al. 1997). This article reviews a variety of family risk factors that contribute to a COA’s likelihood of developing alcoholism and other psychological disorders during adulthood.

## High- and Low-Risk Families

Data from the Epidemiologic Catchment Area Study indicate that 37 percent of people diagnosed with alcohol abuse and/or dependence have a comorbid psychiatric diagnosis, such as antisocial personality disorder (ASPD), bipolar disorder, schizophrenia, or anxiety/ affective disorders (Regier et al. 1990). Findings from the National Comorbidity Study suggest that these rates are substantially higher (i.e., in excess of 50 percent) among alcoholic women (see Kessler et al. 1997). The presence of comorbid psychopathology in the alcoholic parents may increase COAs’ risk for alcoholism and other mental health problems.

To investigate this hypothesis, several studies have attempted to differentiate “high-risk” and “low-risk” families based on the presence of comorbid psychopathological disorders in the alcoholic parents. These studies found that such risk factors were directly related to the COAs’ adjustment. For example, Johnson and Jacob (1995) demonstrated that COA’s with impairments in their behavioral and emotional adjustment were more likely than other COA’s to have parents with more severe alcoholism, comorbid psychopathology, and lower educational levels. Maternal depression in particular predicted higher rates of mental health problems among the COA’s studied. Chassin and colleagues (1991) found that whereas the amount of AOD-related problems among COA’s was predicted by the severity of parental alcohol use, the COAs’ risk for externalizing behavior problems (e.g., aggression and delinquency) depended on the presence of parental comorbid psychiatric diagnoses, such as ASPD and depression.

By differentiating between high-and low-risk alcoholic families, researchers and clinicians can better identify COA’s who are at the highest risk for behavioral and emotional disturbances. Assessment for parental comorbid psychopathology appears to be a useful shorthand for making this differentiation. However, researchers also must determine which family risk factors this shorthand represents. To this end it is necessary to consider all family influences that may affect COA adjustment.

Relevant family influences fall into two categories: (1) alcohol-specific family influences, which selectively predict alcohol abuse and alcoholism, and (2) alcohol-nonspecific family influences, which predict both alcoholism and other psychiatric problems (see table 1). An example of an alcohol-specific family influence is parental modeling of alcohol use as a coping strategy. Alcohol-nonspecific influences include factors such as family violence and physical abuse.

Both alcohol-specific and alcohol-nonspecific processes are part of the network of causal factors (i.e., the etiological matrix) that must be considered when developing models of risk for behavioral and emotional impairment among COA’s. A key aspect of this etiologic matrix is the concept of “nestedness,” which states that in alcoholic families at the highest risk level, multiple co-occurring family risk factors result in an elevated risk for AOD abuse and other mental health problems among the children. These co-occurring influences include severe alcohol dependence and comorbid psychopathology in both parents, high rates of family aggression and violence, and modeling of alcohol use as a means of coping with stress.

It is important to note that although this article focuses on the role of factors in the family “environment” in increasing COAs’ risks, environmental and genetic influences interact with one another in causing psychopathology. The concept of genotype-environment interaction suggests that COA’s with certain “high-risk” genetic makeups (i.e., genotypes) are highly susceptible to behavioral disorders, even under minimal environmental stress. COA’s with “low-risk” genotypes, in contrast, may be susceptible only when exposed to a highly stressful family environment (see article by McGue, pp. 210–217). Accordingly, many models of risk among COA’s use a probabilistic-developmental framework, which proposes that biological/genetic, peer, community, and family influences act in concert (Zucker et al. 1995*a*). Within each of these domains, risk factors can either moderate or mediate COAs’ risk. Moderators affect the strength or direction of the relationship between COA status and maladaptive outcome, whereas mediators are part of the causal relationship between COA status and maladaptive outcome.

## Alcohol-Specific Family Influences on COA Adjustment

Genetic risk factors, such as physiological differences in the sensitivity and reactivity to and the metabolism of alcohol, are an important source of alcohol-specific family influences on COA development (Schuckit 1995). In addition, however, the family plays an important role in shaping a child’s future drinking behavior and attitudes toward alcohol, both through the parents’ behavioral example and through the ways in which the parents filter and interpret societal norms and values regarding alcohol use (Johnson and Pandina 1991). Accordingly, the modeling of parental drinking behavior, the development of alcohol expectancies, and ethnic differences in drinking practices constitute environmental family influences that are related to COAs’ alcohol use.

### Modeling of Parental Drinking Behavior

In alcoholic families, children are routinely exposed to parental alcohol use. Several studies have shown that children and parents tend to exhibit similar drinking practices, indicating that observational learning plays some role in later alcohol use (Webster et al. 1989). Other studies have found that as early as the preschool years, COA’s are more familiar with a wider range of alcoholic beverages and are better able to identify alcoholic beverages by smell than are non-COA’s (Zucker et al. 1995*b*). These observations suggest that more and earlier opportunities for learning about alcohol exist in alcoholic families. However, the pathway from earlier acquisition of information about alcohol to more problematic alcohol use by COA’s has not been well characterized.

Social learning theory suggests that modeling of a behavior such as heavy alcohol use is more likely if the observer (e.g., the child) respects the model (e.g., the parent) (Jacob and Leonard 1994). This modeling hypothesis of alcohol abuse in COA’s is at least partially supported by findings that children of alcoholic fathers are more likely to develop alcoholism themselves if their mothers hold the fathers in high esteem (McCord 1988). The conditions that tend to promote parental modeling (e.g., positive parent-child relations), however, may exist only in certain types of alcoholic families, such as those in which the parent has late-onset and/or less severe alcoholism with little comorbid psychopathology (Jacob and Leonard 1994). Therefore, modeling of parental alcohol use may play an important role in the development of alcoholism only for a subset of COA’s.

### Development of Alcohol Expectancies

Alcohol expectancies are expectations and beliefs regarding the effects of alcohol that are predictive of individual differences in drinking behavior. Reese and colleagues (1994) have proposed that alcohol expectancies serve as mediators that account for how a COA’s internalized observations of parental drinking influence his or her decisions about drinking. Studies comparing alcohol expectancies among COA’s and non-COA’s have found that COA’s appear to have higher expectations that alcohol use will be positive and/or reinforcing (Brown et al. 1987; Sher et al. 1991). Moreover, alcohol expectancies develop as early as during the early school years, well before COA’s are likely to have consumed any significant amounts of alcohol (Zucker et al. 1995*b*; Miller et al. 1990). Taken together, these observations suggest that alcohol expectancies, which are at least partly shaped by early learning experiences in the family, are important in the development of alcohol problems among COA’s.

The relationship between early acquisition of information about alcohol, the development of feelings regarding alcohol use (i.e., affective attributions), and positive or negative alcohol expectancies still is poorly understood. McCord’s (1988) study of children of alcoholic fathers may suggest that the transmission of alcohol problems to the COA is more likely to occur in alcoholic families in which the parents exhibit greater concordance in their drinking patterns and in their values regarding alcohol use (e.g., in homes with two alcoholic parents).

Numerous studies have investigated the utility of the alcohol expectancies concept for explaining how exposure to parental alcohol abuse could lead to elevated rates of alcohol abuse among COA’s. Nevertheless, researchers know little about the relationship between the severity of parental alcoholism and the development of positive or negative expectancies in COA’s.

One study attempting to investigate this relationship has indicated that the more alcohol parents consume, the earlier children acquire alcohol-use schemas (Zucker et al. 1995*b*). To account for such potential associations, future research in this area must consider the variability among alcoholic homes in alcoholism severity (i.e., the amount of alcohol consumed and the kind and extent of drinking-related consequences).

### Ethnicity and Drinking Practices

Although an extensive review of the literature on ethnicity and drinking practices is beyond the scope of this article, it is important to acknowledge that families are embedded in a cultural framework, which has a significant impact on alcohol use. Many studies have shown that people’s beliefs about the effects of alcohol vary depending on their ethnicity (Johnstone 1994). Thus, Christiansen and Teahan (1987) found that Irish adolescents expected fewer social benefits, less sexual prowess, and greater increases in aggressive behavior from alcohol consumption than did American adolescents. Variations in alcohol expectancies are also related to different rates of alcohol use and abuse among different ethnic groups.

Various ethnic or racial groups also differ in the degree to which adolescents rely on parents and family members versus peers as reference groups for acquiring norms regarding alcohol. For example, as compared with Caucasian adolescents, African-American adolescents appear to have more negative expectancies about alcohol use and to regard parental disapproval of alcohol use as more important (Johnstone 1994). Given the strong relationship between ethnicity and alcohol-specific family risk factors, more research on cultural factors that affect the risk for alcoholism in the COA population is clearly warranted.

## Alcohol-Nonspecific Family Influences on COA Adjustment

Like alcohol-specific family influences, alcohol-nonspecific family influences on COA development include heritable and environmental risk factors. An example of a heritable alcohol-nonspecific family risk factor is biobehavioral dysregulation, which may manifest itself at the physiological level as an increased reactivity of the autonomic nervous system to stressful stimuli (e.g., more easily elevated heart rate) or at the behavioral level as a “difficult temperament” (e.g., hyperactivity and high emotional reactivity). Environmental alcohol-nonspecific family risk factors include characteristics such as parental psychopathology; socioeconomic status (SES); general family psychopathology, including impaired family interactional patterns, family aggression and violence; and impaired parental cognitive abilities.

### Parental Psychopathology

As discussed earlier in this article, a significant proportion of COA’s are reared in families with parents who suffer from psychiatric disorders in addition to alcohol abuse or dependence. Research in other mental health areas has long established a link between parental psychopathology and child mental health problems. For example, parental ASPD has been associated with conduct disorder in children, and parental depression has been linked to depression in children. Alcohol researchers, however, are just beginning to acknowledge such an association (Sher et al. 1991; Chassin et al. 1991; Zucker et al. 1995*a*).

Several studies that have classified alcoholic families based on the presence of comorbid parental psychopathology have indicated that this alcohol-nonspecific risk factor may account for elevated rates of non-alcohol-related problems, such as delinquency and depression, among COA’s (Johnson and Jacob 1995; Chassin et al. 1991). In addition, in many heavily troubled alcoholic families, in which alcoholism coexists with other parental mental health problems, both parents, rather than just one, suffer from psychiatric disturbances (Johnson and Jacob 1995). Consequently, children in this subset of alcoholic families are less likely to experience the protective effects of having one parent who can provide appropriate nurturance and discipline. This concept of aggregation of risk factors is further discussed later in this article.

### Family SES

Various types of psychopathology, including AOD use disorders, are known to be associated with economic disadvantage (Robins and Regier 1991). Thus, the rates of AOD abuse are significantly higher among families with lower SES than among families with higher SES. It is possible, however, that low SES is not in itself a risk factor, but is a proxy variable for other individual and/or family risk factors. In fact, recent studies of alcoholic families suggest that low SES does not in itself account for child psychosocial impairment, but is only one of a network of risk variables to which some COA’s are exposed (Zucker et al. 1996*b*). Although the influence of poverty and its accompanying stresses on child development should not be underestimated, sociodemographic factors, such as SES, appear to be linked to numerous other influences, some of which may predict mental health outcomes of COA’s more directly.

### General Family Psychopathology

#### Impaired Family Interactional Patterns

Numerous studies have documented that COA’s grow up in rearing environments that generally are characterized by low levels of cohesion (i.e., no close bonds among family members) and high levels of conflict (West and Prinz 1987; Sher 1991; Seilhamer and Jacob 1990). In turn, high levels of family conflict predict adolescent alcohol problems (Johnson and Pandina 1991). Although early observational studies of alcoholic families also suggested that the level of family cohesion depended on whether the alcoholic parent was actively drinking (Steinglass 1979), this link between the alcoholic’s drinking status and the family’s interactional style has not been demonstrated unequivocally.

Because family systems grow and change over time, different children within an alcoholic family may be differentially affected by various stages of parental alcoholism and the related shifts in family functioning. For example, Puttler and colleagues (1996) observed that COA’s whose alcoholic parents were in remission did not differ in behavioral and cognitive functioning from their non-COA peers, whereas children of active alcoholics performed less well. The concept of shared and nonshared environmental influences may account for these differences (see the article by McGue, pp. 210–217). Shared environmental effects, such as consistent aspects of parenting style (e.g., a tendency toward authoritarian or punitive parenting), are relatively stable across the family’s “life cycle” and impact all children in the family equally. Conversely, nonshared environmental effects differ for each child in a family and may depend on the child’s age and birth position as well as on the life-cycle phase in which the family finds itself at crucial points during the child’s development. For example, a period during which an alcoholic parent sustains heavy drinking that takes him out of the home on a nightly basis and which results in a drunk-driving arrest will affect a 12-year-old child and his or her 3-year-old sibling differently.

Wolin and colleagues (1980) have proposed that alcoholic families fall into two groups—subsumptive and distinctive—based on the effect that parental alcohol use has on their daily routines and interactions. In subsumptive families, parental alcohol use substantially alters the family’s routines (e.g., common mealtimes) and rituals (e.g., the way holidays are celebrated). Conversely, in distinctive families, parental alcohol use does not affect family routines and rituals. Wolin and colleagues (1980) have found that COA’s from distinctive families were less likely to develop alcohol problems than were COA’s from subsumptive families. Although the distinction between subsumptive and distinctive families may also be a marker for other family risk factors, such as more severe parental alcoholism, this study demonstrates how global concepts, such as family cohesion, can be expressed empirically as measurable variables (e.g., whether or not a family celebrates holidays together), which can then be linked to COA outcome.

Other researchers also have investigated the interactional patterns of alcoholic families. For example, one series of studies found that compared with nonalcoholic families, alcoholic families demonstrated poorer problem-solving abilities, both among the parents and within the family as a whole (Jacob and Leonard 1994). These poor communication and problem-solving skills may be mechanisms through which lack of cohesion and increased conflict develop and escalate in alcoholic families.

Given the high rates of problematic family interactions, it is not surprising that alcoholic families are at increased risk of divorce. This family disruption can have further negative impact on COA’s and their development—for example, by increasing the likelihood of reduced family income, exposing COA’s to custody disputes, and continuing or escalating parental conflict over child-rearing practices. It is difficult, however, to separate the direct impact of divorce on COA’s from the impact of other family risk factors. For example, predivorce marital conflict not only affects the likelihood that a divorce will occur, but also is predictive of lower levels of parental monitoring of the children and poorer affective bonds between parents and children. These factors, in turn, are associated with poor psychological outcome and earlier AOD involvement of the children (Wills et al. 1996).

#### Family Aggression and Violence

Although numerous studies have demonstrated an association between alcoholism and spousal violence, many of these studies suffer from lack of methodological rigor (e.g., they lack control groups or they oversample population groups that likely have high rates of both violence and alcoholism) (Sher 1991). Other investigations have suggested that a link between alcoholism in a parent and increased family aggression and/or child abuse is particularly characteristic of families of alcoholics with comorbid ASPD (Zucker et al. 1996*b*). In these alcoholic families, in which parents demonstrate high rates of deviant and aggressive behaviors, frequent exposure to family violence may increase the risk for aggression among the children. Early childhood aggression, in turn, is known to be related to the development of later conduct problems and delinquency. Moreover, childhood aggression is more closely linked to the development of a severe, early onset form of alcoholism than is general behavioral deviancy (Jaffe et al. 1988). Consequently, COA’s from families with comorbid ASPD have a substantially increased likelihood of experiencing a variety of early and severe externalizing behavior problems, including experimentation with and abuse of alcohol; disregard for authority figures, such as parents and teachers; and trouble with the law.

### Parental Cognitive Impairment

The negative effects of alcohol on cognitive functioning in heavy drinkers are well documented (Ron 1987). In addition, recent studies suggest that poor performance by alcoholics on neuropsychological tests may not only result from alcohol’s neurotoxicity, but also may reflect premorbid cognitive deficits (Sher et al. 1991). Consequently, COA’s may be raised by parents with relatively poorer cognitive abilities than children reared by nonalcoholic parents. Furthermore, a strong correlation exists between the cognitive abilities of parents and their children. Accordingly, a lack of stimulation in the rearing environment may account in part for the pattern of cognitive impairments, lower academic achievement, and increased school failure found in COA’s compared with non-COA’s (Johnson and Rolf 1988). In keeping with this hypothesis, Noll and colleagues (1992) found that preschool-aged COA’s exhibited poorer language and reasoning skills than did non-COA’s and that poorer performance among the COA’s was predicted by the lower quality of stimulation present in the home. Poor academic achievement and school failure, in turn, not only place COA’s at risk for lower educational attainment, but also may act synergistically with early behavior problems to bring COA’s into contact with a more deviant peer group (Dishion et al. 1991), thereby increasing their risk for externalizing behavior problems and subsequent alcohol abuse.

## Aggregation of Risk Factors

The various alcohol-specific and alcohol-nonspecific risk factors described in this article do not exist in isolation. Instead, a recent study has demonstrated that by subtyping alcoholic families based on the presence or absence of ASPD in the alcoholic parent, it is possible to identify highly troubled families in whom a variety of risk factors aggregate (Zucker et al. 1996*b*) (see table 2). In this study, alcoholic fathers were classified as either antisocial or nonantisocial alcoholics. The families of antisocial alcoholics and nonantisocial alcoholics were then compared with control families in which neither parent had an AOD use disorder or ASPD. Compared with the other two groups of families, the antisocial alcoholic families had the following characteristics:

- Both fathers and mothers had the highest rates of antisociality, depression, and more severe alcohol problems.
- The parents had the lowest intellectual abilities.
- The parents were most likely to display verbally and physically abusive behavior toward each other and to use aggressive disciplinary practices (e.g., spanking) with their children.
- The families had the densest family history of alcoholism, suggesting that children in these families were more likely to be exposed to multiple extended family members who abused alcohol and to have a higher genetic risk for alcoholism.
- The families had the lowest SES. The SES in itself, however, did not account for the other group differences.

Nonantisocial alcoholic families formed an intermediate group that did not differ from control families on several risk variables, including measures of family aggression and violence.

Not surprisingly, compared with the control children, the preschool-aged children of antisocial alcoholics showed substantially elevated rates of clinically significant behavior problems. Thus, almost one-fifth of these children demonstrated externalizing behavior problems (e.g., aggression and oppositionality), and one-tenth exhibited internalizing behavior problems (e.g., anxiety and depression). The children of nonantisocial alcoholics, in contrast, did not differ from the control children. Given the young age of the children and the fact that they did not yet use alcohol, it is not possible to assess whether the family risk variables analyzed are associated with future alcohol use and abuse in COA’s. The findings suggest, however, that the children of antisocial alcoholics may already exhibit the initial stages of a pathway into early onset alcohol problems that is associated with aggression and conduct disorders (Zucker and Gomberg 1986).

The findings of the study by Zucker and colleagues (1996*b*) have demonstrated that antisocial alcoholic families are more impaired on measures of antisociality, depression, intellectual abilities, violence and aggression, and SES than are families of nonantisocial alcoholics, who, in turn, are more impaired than nonalcoholic families. One must ask, however, whether these characteristics are consequences of the father’s ASPD or whether they are perhaps attributable to other factors. In other words, it is essential to determine if the father’s ASPD diagnosis is a stand-alone indicator that by itself can sufficiently explain the outcomes of the family and of the children or if it is only a marker for a set of multifactorial, co-occurring risk processes in the family. The findings of several analyses have favored the latter explanation, as follows (Zucker et al. 1996*a*, 1996*b*):

- Family risk factors generally co-aggregate in antisocial alcoholic families.
- Family risk factors in antisocial alcoholic families often exhibit the characteristic of a statistical “type” (i.e., they co-occur in these families significantly more often than in the general population).
- None of the family risk factors, including paternal ASPD, by itself is sufficient to explain the differences among families of antisocial alcoholics, nonantisocial alcoholics, and control subjects.
- Some of the family risk factors appear to have developed over time (e.g., the antisocial alcoholic fathers’ SES has declined between childhood and adulthood), suggesting the operation of an ongoing process.

To explain these observations, Zucker and colleagues (1995*a*) have developed the nestedness concept, which states that among some subtypes of alcoholic families, risk factors aggregate in a nonrandom manner.

Findings such as the ones just described demonstrate that the heterogeneity in outcome among COA’s likely results from differences among alcoholic families in the density of family risk factors. Another conclusion from these studies is that researchers and clinicians can establish markers that allow them to identify the most damaging family environments. One of the major tenets of developmental psychopathology is that the developmental pathways of children naturally lead toward positive adaptation and that only under repeated stress or insult does sustained psychopathology occur (Cicchetti and Cohen 1995). Consequently, whereas most COA’s will exhibit positive adult adaptation, the likelihood of a positive outcome will decrease for COA’s from families in which multiple risk factors occur in concert and in which the operation of resiliency factors is more restricted.

The identification of such high-risk family environments is important for designing appropriate interventions. For example, it appears plausible that standard prevention measures targeted at COA’s are easiest to implement among lower risk families (e.g., those without comorbid ASPD), in whom risk factors are more likely to be malleable. Children of antisocial alcoholics, on the other hand, may benefit most from more complex, multisystemic prevention and/or treatment strategies that simultaneously intervene at the level of the individual COA, the marital dyad, the family, and the community (e.g., schools and peer systems).

## Conclusions

Researchers have identified many family variables that differentiate alcoholic families from nonalcoholic families and that putatively place COA’s at risk for the development of alcoholism and other mental health problems. However, a need still exists for studies that assess both alcohol-specific and alcohol-nonspecific risk factors when tracking COA outcomes, because these two types of risk factors may play differential roles in the development of various types of alcoholism among COA’s. Moreover, future research must more accurately determine the developmental point at which the influences of alcohol-specific and alcohol-nonspecific processes on COA’s begin to converge. Such analyses must be performed in COA’s, because in alcoholics the two processes have long since merged and are both driving the continued problematic alcohol use (Zucker et al. 1995*a*). Future studies must also take into consideration that various risk factors may exert their greatest effects on COA’s at different times during development. For example, poor family problem-solving skills may be most detrimental to COA’s in their teens, a developmental period when negotiation and compromise between parents and children are important. Long-term (i.e., longitudinal) studies of COA’s ultimately will allow researchers to address such issues. The concept of nestedness implies that many risk factors aggregate in high-risk families. The use of more sophisticated research designs and statistical techniques will allow investigators to identify those risk factors that are most important in the development of alcoholism and other mental health problems among COA’s. Such analyses can also improve the design of appropriate prevention and intervention efforts. Finally, given the ever-increasing information on heterogeneity among alcoholic families, alcohol researchers not only must begin to routinely account for this variability in the design of their studies, but they also must develop an improved vocabulary to describe such differences.

## Figures and Tables

**Table 1:** Family Risk Factors Affecting the Development of Psychopathology Among Children of Alcoholics (COA’s) Compared With Children of Nonalcoholics

Risk Factor	Research Findings
**Alcohol-Specific Family Influences**^1^	
• Modeling of drinking behavior	COA’s are more familiar with a wider range of alcoholic beverages at a younger age and develop alcohol-use schemas (i.e., experience-based beliefs) earlier.
• Alcohol expectancies	COA’s have more positive expectancies regarding the reinforcing value of alcohol (i.e., they are more likely to expect that alcohol will make them feel good).
• Ethnicity and drinking practices	COA’s from certain ethnic groups may be at increased risk for alcohol abuse because of the interaction between alcohol expectancies and ethnicity.
**Alcohol-Nonspecific Family Influences**^1^	
• Parent psychopathology	Certain subgroups of COA’s are raised in families in which parents have psychiatric disturbances, such as antisocial personality disorder or depression, in addition to alcohol dependence.
• Socioeconomic status (SES)	COA’s are more likely to come from lower SES homes in which the families are exposed to financial stress.
• General family psychopathology	Alcoholic families are characterized by low cohesion (i.e., little closeness among family members), high conflict, and poor problem-solving skills. COA’s are more likely to come from broken homes.
• Family aggression/violence	COA’s may be more likely to be the targets of physical abuse and to witness family violence.
• Parental cognitive impairment	COA’s are more likely to be raised by parents with poorer cognitive abilities and in an environment lacking stimulation.

1 Alcohol-specific family influences selectively predict alcohol abuse and dependence, whereas alcohol-nonspecific family influences predict a variety of psychiatric problems including alcoholism.

**Table 2:** Aggregation of Risk Factors in Alcoholic Families: High-Risk Versus Low-Risk Family Environments

Child Risk Factor	High-Risk Environment	Low-Risk Environment
Parental psychopathology	Alcoholic parent has comorbid psychopathology	Alcoholic parent has alcoholism without comorbid psychology
Assortative mating^1^	Both parents are likely affected by alcohol and other drug abuse and/or other psychopathology	Generally only one parent is affected by alcohol and other drug abuse
Alcohol use	More severe and/or problematic	Less severe and/or problematic
Parental intellect	Lower	Higher
Family aggression	High rates of aggression toward child and violence between parents	Low rates of family aggression and violence
Family socioeconomic status	Lower	Higher

1 The tendency among people to choose a partner who has similar characteristics or traits to one’s self (e.g., alcohol drinking patterns).

## Footnotes

1 Terms such as “alcohol abuse,” “alcohol problems,” “alcoholism,” and “problem drinking” often are used interchangeably in the literature. In this article, these terms are therefore used as they were in the original studies cited.
